# Coloration and Chromatic Sensing Behavior of Electrospun Cellulose Fibers with Curcumin

**DOI:** 10.3390/nano11010222

**Published:** 2021-01-16

**Authors:** Minhee Kim, Hoik Lee, Myungwoong Kim, Yoon Cheol Park

**Affiliations:** 1 Korea Institute of Industrial Technology, 143, Hanggaulro, Sangnok-gu, Ansan-si 15588, Gyeonggi-do, Korea; chatelier@kitech.re.kr; 2 Department of Chemistry and Chemical Engineering, Inha University, Incheon 22212, Korea

**Keywords:** cellulose fiber, coloration, curcumin, electrospinning, chromatic sensor

## Abstract

The effective approach for coloration and chromatic sensing of electrospun cellulose fabrics with a natural colorant, curcumin, is demonstrated. To achieve high surface area, the morphology of fiber was controlled to have rough and porous surface through an electrospinning of a cellulose acetate (CA) solution under optimized electrospinning parameters and solvent system. The resulting CA fibers were treated with a curcumin dye/NaOH ethanol solution, in which deacetylation of the CA fiber and high-quality coloration with curcumin were simultaneously achieved. As a control, a cotton fiber with similar diameter and smooth surface morphology was treated by the same method, resulting in poor coloration quality. The difference can be attributed to high surface area as well as trapping of dye molecules inside of cellulose fiber during deacetylation. Both fibers were further utilized for a chromatic sensing application for specific toxic gases. The incorporated curcumin dye responded to hydrogen chloride and ammonia gases reversibly via keto-enol tautomerism, and, as a consequence, the color was reversibly changed between reddish-brown and yellow colors. The cellulose fiber fabricated by the electrospinning showed ten times higher and two times quicker responsiveness compared to curcumin-colored cotton fiber sample prepared with the same immersion method.

## 1. Introduction

Cellulose is one of the promising materials as a sustainable and renewable source due to its great abundance on Earth [1]. It is composed of linear glucose polymer chains that form a number of inter- and intra-molecular hydrogen bonds in its macromolecular structure. The strong hydrogen bonding allows exhibiting excellent thermal stability and chemical resistance [2]. Generally, cellulose has been used as a raw material for fabrics such as cotton, jute, and flax for a long time, allowing its use for a broad range of applications in textiles industry [3]. Furthermore, cellulose has been a potential candidate material for other various significant applications such as biosensors, scaffolds, packaging films, and membranes.

Dyeing is an important process in the textile industry, and the dyeing technology has been evolved towards the improvement of aesthetic properties of the apparel [4]. For effective dyeing process, many parameters should be considered. The interaction between a dye molecule and a fabric as well as the diffusion of dye molecules into the internal part of the fabric are essential. Therefore, the dyeing properties of fabrics are largely affected by the macroscopic structure of fabrics as well as the microscopic structures at a molecular level. Moreover, one of the obvious features to affect the dyeing behavior is the specific surface area of a fabric. It is well known that a finer structure leads to greater dyeing rate [5]. The dye itself is also highly significant to the dyeing process. Up to now, synthetic dyes have been widely utilized for dyeing textiles; however, they often exhibit toxicity which negatively affects our environment. Therefore, the interests in natural dyes, obtained from various natural sources such as plants, insects, and minerals, have increased significantly due to their low toxicity and allergic effects [6]. Among a variety of natural dyes, curcumin, a naturally occurring compound in the plant *Curcuma longa* L., is widely utilized material as a spice and coloring agent for food over the world [7]. More importantly, it has been employed as a non-toxic and eco-friendly functional materials platform for diverse applications in the fields of biomedical materials and colorimetric sensors [8,9,10].

Generally, dyeing behavior is highly dependent on the reaction conditions such as temperature, pH, and dye concentration, and the molecular structure of the dye affects the diffusion of dye molecules [11]. Particularly for dyeing cellulose fabrics, processing under harsh conditions for dye fixation is often required, for example, the process with reactive dyes in a highly basic medium at increased temperature. Herein, we demonstrate a simple but highly effective approach for dyeing cellulose fabrics without processing under known essential dyeing conditions. To do so, we fabricated cellulose acetate (CA) fibers exhibiting rough and porous surface morphology to provide high surface area via an electrospinning technique [12], which is a powerful tool to fabricate a continuous non-woven fabric with a fine structure with controlled fiber diameter that can be readily adjusted with a systematic variation of electrospinning parameters such as polymer concentration, voltage, and tip-to-collector distance [13]. Since cellulose is challenging to dissolve in common solvent systems due to the tremendous amount of hydroxyl group capable of the formation of inter- and intra-chain hydrogen bonds, CA fiber was fabricated through electrospinning. Fabricated CA fibers with a porous surface structure were deacetylated [12,14] and dyed with curcumin simultaneously by a simple immersion method, which was found to be ineffective for dyeing cellulose cotton fiber fabric with smooth surface morphology. The adsorbed curcumin dye on the cellulose fiber was stable upon repetitive washings with polar solvents. The resulting curcumin-dyed cellulose fiber was further utilized for sensing toxic ammonia and hydrogen chloride vapors that induce the change in a conjugation state of curcumin dye, and, therefore, clear and reversible color changes observable by the naked eye were attained, though the dyed-cotton fiber was not effective. These studies offer a simple but versatile dyeing strategy with a natural dye on cellulose fabrics that can be further applicable to chromatic sensory platforms for hazardous gaseous species.

## 2. Materials and Methods

### 2.1. Materials

Curcumin (Cur, from curcuma longa (Turmeric)), cellulose acetate (CA, Mn~50,000 g/mol), acetone (≥99.5%), and hydrochloric acid (HCl, ACS reagent, 37%) were purchased from Sigma-Aldrich Co. Ltd. (Milwaukee, WI, USA). Cotton (ISO ADJ COTTON 1602002) was purchased from Testfabrics Korea, Inc. (Ansan, Korea). Ammonia solution (28–30 wt%) was obtained from Junsei Chemical, Co., Ltd. (Tokyo, Japan). Ethyl alcohol (anhydrous, 99.8%) and sodium hydroxide standard solution (1 N) were purchased from Daejung Chemicals & Metals Co., Ltd. (Siheung, Korea). Dichloromethane (DCM) was obtained Alfa Aesar (Ward Hill, MA, USA). Deionized water (DI water) was obtained using Milli-Q system. Unless noted, the reagents were used without further purification.

### 2.2. Characterization

Field emission scanning electron microscopy (FE-SEM, SU-8010, Hitachi Ltd., Japan) was utilized to study morphology and size of cellulose fibers. All SEM specimens were coated with osmium using a JFC-1200 fin coater (JEOL, Tokyo, Japan) for 60 s prior to observation to make the samples conductive. Fourier transform infrared (FT-IR; Thermo Nicolet NEXUS 670 FT-IR spectrometer, Thermo Fisher Scientific, Massachusetts, USA) spectroscopy was used to study chemical structures of cellulose acetate fibers over the wavenumber range from 4000 to 400 cm^−1^.

### 2.3. Coloration Process for Cellulose Fabrics with Curcumin

Cellulose fiber samples were prepared through the deacetylation of cellulose acetate fiber samples, which were fabricated via an electrospinning process. The electrospinning apparatus (ESR200RD, NanoNC, Korea) consisted of a 30 kV voltage generator, a metallic drum collector (NNC-DC90H, NanoNC, Seoul, Korea), and a syringe pump allowing to adjust the injection rate of the polymer solution. The processing parameters for the electrospinning, i.e., applied voltage, tip-to-collector distance, and flow rate of solution, were set to 15 kV, 10 cm, and 1.0 mL/h, respectively. The electrospinning experiments were conducted at room temperature with a humidity of approximately 40%. Cellulose acetate powder was dissolved to make 10 wt% solution in DMF/acetone mixed solution (1/2 *v/v*), followed by stirring at room temperature for 8 h. The resulting viscous colorless and transparent polymer solution was then utilized for electrospinning. A plastic syringe equipped with a capillary tip (inner diameter: 0.6 mm) was used to inject a polymer solution into the electrospinning apparatus. The CA fiber mat samples (3 cm × 3 cm) were subjected to the immersion in 10 mL of curcumin/NaOH solution in ethyl alcohol (5 mM for curcumin, 50 mM for NaOH) at room temperature for 24 h, followed by drying at 60 °C for deacetylation of CA fabric and subsequent dyeing of the cellulose fabric. As a control, cellulose cotton fabric (3 cm × 3 cm) was prepared and immersed in 10 mL of curcumin solution (5 mM in ethanol) for 24 h and subsequently dried at 60 °C.

### 2.4. Chromatic Sensing Experiment

**Figure 1:**
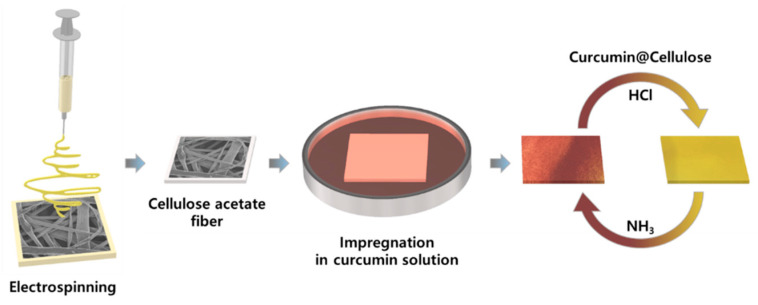
Schematic illustration showing the fabrication process of fabric samples dyed with curcumin from cellulose acetate and its chromatic responsive behavior upon the exposure to hydrogen chloride and ammonia gas.

The curcumin incorporated cellulose fabric and cotton fabric samples were cut into square shape (1 cm × 1 cm). The prepared specimens were placed in a customized gas chamber for testing ammonia and hydrogen chloride gas detection. For saturation of ammonia gas in the chamber, the plate where 40 ppm ammonia solution was dropped was placed in the testing chamber, followed by heating with a heat gun. A peristaltic pump system (RP-1100, Tokyo Co., LTD, Tokyo, Japan) was connected to the chamber and was used to remove the ammonia gas. The ammonia gas was exposed directly to the fabric samples for 90 s. Induced color changes were monitored every 3 s with computer color matching system (CE-7000A, X-rite, Grand Rapids, MI, USA). Upon the exposure to ammonia gas, the chamber was saturated with hydrogen chloride vapor by evaporation from hydrochloric acid aqueous solution for three days. The color changes in the fabric samples were then monitored in the same manner. Figure 1 shows the scheme for fabrication and dyeing process of cellulose fiber mat and its reversible responsiveness upon ammonia and hydrogen chloride vapors.

## 3. Results and Discussion

### 3.1. Morphologies of Cotton Fibers and Electrospun CA Fibers

**Figure 2:**
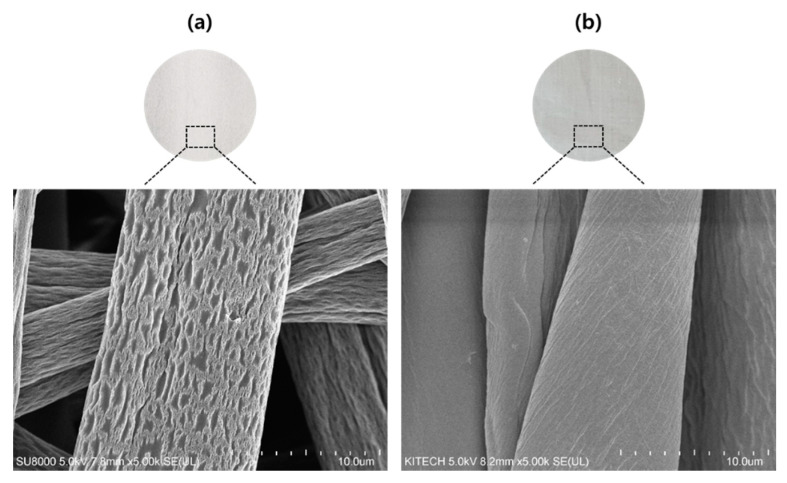
SEM images showing morphologies of: (**a**) electrospun cellulose acetate fiber; and (**b**) cotton fabric.

Morphologies of CA fibers and cotton fibers were examined using FE-SEM, and the resulting images are shown in Figure 2. The surface morphology of CA fiber was observed to be rough and porous, while the surface of cotton fabric was highly smooth. It has been reported that CA fiber can be easily fabricated with a smooth surface similar to the cotton fabric via electrospinning process [12] The control of surface morphology of electrospun CA fibers can be achieved by adjusting solvent system [15,16,17]. Typically, electrospun fibers fabricated with a good solvent system to polymer shows a very smooth surface morphology due to the homogeneity of polymer solution. However, in a poor solvent system, polymer chains tend to interact with other polymer chains rather than solvent molecules, which limits homogeneous diffusion behavior of polymer chains during the flight when electrospinning is performed, consequently making a porous and rough morphology. In addition, the use of solvents exhibiting high vapor pressure also results in the rough surface. Hence, the diffusion of the polymer chains during the flight of polymer filaments as well as the intrinsic properties of the solvent are the key parameters governing the morphology of electrospun fiber [18].

We employed a DCM/acetone mixed system (2/1 *v/v*) to attain fibrous structure of CA via electrospinning, which facilitates a porous morphology on the fiber surface in submicron scale. The concentration of CA in the solution was fixed at 10 wt% in order to gain a uniform fiber, as we observed the degradation of the spinnability showing a bead structure in resulting samples at the solution concentration less than 10 wt%. Under these processing conditions, the diameter of electrospun CA fiber was comparable to that of cotton fiber. Therefore, the CA fiber and cotton fiber samples show the difference in the porosity and surface area, which could play an important role in their dyeing behaviors. The original color of electrospun CA fiber and cotton fabric is almost identical; both samples show a grey color (Figure 2).

### 3.2. Coloration Behavior of Cellulose Fabric with Curcumin

**Figure 3:**
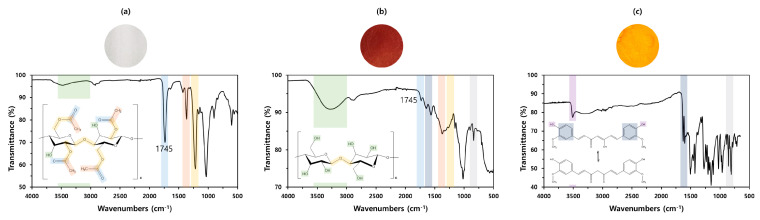
FT-IR spectra of: (**a**) electrospun CA fiber sample; (**b**) cellulose fiber sample treated by the immersion in curcumin/NaOH ethanol solution; and (**c**) curcumin.

The fiber samples were subjected to dyeing process, which was achieved by a simple immersion of both samples in a solution of curcumin (5 mM) and NaOH (50 mM) in ethanol. This process plays dual roles for CA fiber: (i) deacetylation of CA fiber by a strong base (NaOH) to form cellulose fiber [19]; and (ii) incorporation of curcumin dye in cellulose fiber (Figure S1a). The use of NaOH for the deacetylation is beneficial, as it can be used in different solvent systems, e.g., water and alcohol [20]. During the deacetylation process, hydroxide ions diffuse into the CA fiber, leading to the cleavage of acetyl group in the CA fiber [19,20,21,22,23] The deacetylation reaction was clearly confirmed by FT-IR spectroscopy. As shown in Figure 3a, cellulose acetate fiber exhibited absorption peaks at 1745, 1375, and 1235 cm^−1^, which are assigned to C=O and C-O in acetyl group and C-O group in cellulose components, respectively [14,24]. The characteristic peaks of the acetyl group almost disappeared upon the immersion process, indicating successful formation of cellulose fiber. In addition, the intensity of the peak at 3000–3500 cm^−1^ assigned to O-H stretching was observed to be stronger and broader in cellulose fiber than those in cellulose acetate fiber. This intensity increase is due to a larger amount of alcohol functionality in cellulose compared to CA. Upon the immersion process, the characteristic peaks from curcumin dye were also observed from the dyed cellulose fiber sample at 856, 1153, 1281, 1510, 1602, and 1627 cm^−1^ [25], suggesting the immersion process was equally effective to incorporate dye molecules in the cellulose fiber.

**Figure 4:**
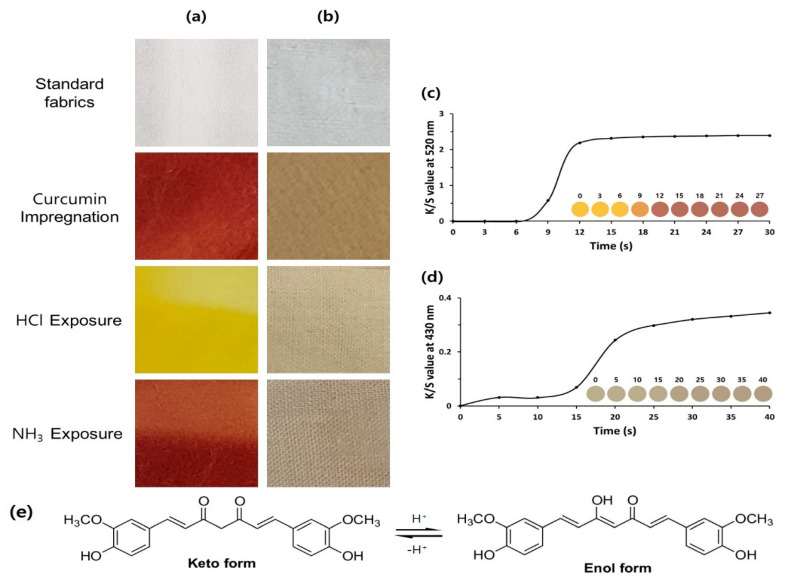
The photographs showing the color changes in (**a**) CECF and (**b**) dyed cotton samples; the traced K/S value as a function of time of (**c**) CECF and (**d**) dyed cotton samples; and (**e**) the scheme for the reaction responsible for the color change.

After the immersion process, both samples exhibited completely different colors, and the difference of dyeing behaviors for both fibers was readily observed even with the naked eye. The color of the electrospun CA fiber sample changed from white to reddish-brown. However, cotton fiber sample showed dark ivory color upon the immersion process (top images in Figure 2 and Figure 4) The original color of the curcumin solution at neutral pH is orange; however, the color changes to reddish-brown in basic solution, which is what was observed in curcumin-dyed deacetylated cellulose fiber (CECF) sample. Cotton fiber did not show the reddish-brown color, indicating poor interaction of curcumin molecule to the smooth surface. This result suggests that curcumin molecules particularly strongly interact with the surface of CECF so that the color visibility is highly enhanced.

Since the cotton fiber is also comprised of cellulose, the difference of the CECF from the cotton fiber is the surface morphology and the deacetylation process. First, the rough surface, i.e., high surface area, can provide more adsorption sites for curcumin dye molecules than smooth surface. The dye molecules can have more chances to interact with the surface, and, hence, the dye adsorption can be enhanced. Secondly, during the immersion process for dyeing, deacetylation simultaneously occurs. The volume occupied by acetyl group diminishes during the deacetylation process; consequently, resulting cellulose chains begin to interact with each other to form semi-crystalline structure [26]. Meanwhile, the curcumin molecule also adsorbs on the surface, and, by chance, the dye molecules diffuse into the free volume generated by deacetylation and are trapped by cellulose chains near the surface. The color of CECF did not significantly change with repetitive washing with protic solvents such as water and ethanol, suggesting that the curcumin molecules, which are not expected to react with the chemical functionality in cellulose, are strongly adsorbed on the surface of CECF (Figures S2 and S3). It is worth noting that it is widely accepted that the temperature control and the use of reactive dyes are crucial for dyeing of cellulose fabric such as cotton. Our results suggest that dyeing of the electrospun CA fibers can be easily accomplished by the simple immersion method under mild conditions without using any synthetic dyes.

### 3.3. Chromatic Gas Sensing Behaviors

Enhanced color visibility of the CECF enables us to expand the applicability of cellulose fiber, e.g., chromatic detection of toxic gas molecules such as NH_3_ and HCl gases. Figure 4 and Figure S4 shows the color transition behaviors of the CECF and curcumin-dyed cotton fabric samples upon exposure to HCl and NH_3_ gases. The color transition of curcumin is attributed to keto-enol tautomerism, which can be easily controlled by the pH in the environment [27,28]. Under basic condition, the keto tautomer is changed into the state comprising the mixture of enol and enolate forms, and, as a consequence, dramatic color change from yellow to reddish-brown is observed. In the case of CECF, the reddish-brown color was transformed to yellow upon exposure to HCl vapor, leading to the state where the keto formation is favored [29]. Subsequent change of the environment with NH_3_ vapor made quick recovery to its original color in a few seconds, highlighting the reversible nature of the tautomerism (Figure 4a). The color transition by NH_3_ vapor was observed during 30 s of exposure by examining K/S value, which indicates the depth of the color of dyed fabric (Figure 4c). The color change in CECF was completed within 12 s, obtained by the analysis of peak intensity at the 520 nm (Figure S4).

The degree of color change in CECF was observed to be approximately ten times higher than that in dyed cotton fiber, which is the results of different dyeing behavior. As mentioned above, the color transition in CECF occurs clearly upon the changes in the pH of the environment; however, the cotton fabric did not exhibit a distinct color change, as shown in Figure 4b. The color of cotton fabric changed faintly from dark ivory to ivory upon the exposure to HCl vapor, and it was changed to slight dark ivory color after the exposure to NH_3_ vapor, although it was not clearly distinguished with the naked eye (Figure 4b). The color change in the dyed cotton fabric was identified by K/S value obtained by tracing the change of peak intensity at 430 nm (Figure 4d). The color transition was complete in 25 s, which is approximately two times slower than CECF.

## 4. Conclusions

We demonstrated the strategy to perform dyeing process for cellulose fabrics that does not require any harsh processing conditions. The morphology of CA fibers was controlled using an electrospinning technique to exhibit rough and porous surface. The diameter of the CA fibers was also controlled to be similar to that of the fiber in commercially available cotton fabrics. The electrospun CA fiber and cotton fabric samples were immersed in curcumin/NaOH ethanol solution for dyeing. The electrospun CA fiber was deacetylated to form curcumin-incorporated cellulose fiber, although the cotton fabric was not effectively dyed under the same conditions. The color of CECF was not removed upon repetitive washing process, strongly suggesting that the proposed approach with the CA fibers exhibiting rough surface is highly effective to impregnate curcumin dye molecules into cellulose fiber. The CECF was further applied as a specific gas-responsive material platform, allowing to sense toxic gases. The conjugation state of the incorporated curcumin dye molecule was reversibly changed via keto-enol tautomerism with protonation and deprotonation reactions by HCl and NH_3_ vapors, and, therefore, the color was accordingly changed. The color of CFCE was rapidly changed (~12 s) and clearly observable with the naked eye, which is an approximately two-fold faster and ten-fold higher color signal intensity compared to the curcumin-incorporated cotton fiber sample prepared with the same immersion method. It is possible that the methodology here will further be expanded into other polymeric nanofibrous materials with the control of surface morphology in nanoscales.

## Data Availability

The data presented in this study are available on request from the corresponding author.

## Supplementary Materials

The following are available online at https://www.mdpi.com/2079-4991/11/1/222/s1, Figure S1: SEM images showing morphologies of fabricated fibers; Figure S2: K/S value plots and SEM images of CECFs before and after washing process; Figure S3: Photographs showing fastness test results for CECFs upon exposure to HCl vapor before and after washing process; Figure S4: Plots showing the changes of K/S value upon the exposure of fabric samples to ammonia gas.
