# Supplementary material for: Coloration and Chromatic Sensing Behavior of Electrospun Cellulose Fibers with Curcumin
Source: Nanomaterials (Basel). 2021 Jan 16;11(1):222. doi: 10.3390/nano11010222 (PMC7830206; doi:10.3390/nano11010222)
Supplement: Supplementary file 1 [file nanomaterials-11-00222-s001.pdf]

nanomaterials

Supporting Information

Coloration and Chromatic Sensing Behavior of Electrospun Cellulose Fibers with Curcumin

Minhee Kim [1], Hoik Lee [1,*], Myungwoong Kim [2,*] and Yoon Cheol Park [1,*]

[1] Korea Institute of Industrial Technology, 143, Hanggaulro, Sangnok-gu, Ansan-si, Gyeonggi-do, 15588, Republic of Korea
[2] Department of Chemistry and Chemical Engineering, Inha University, Incheon 22212, Republic of Korea
* Correspondence: hoik@kitech.re.kr (H.L), mkim233@inha.ac.kr (M.K.), ycpark@kitech.re.kr (Y.C.P.)

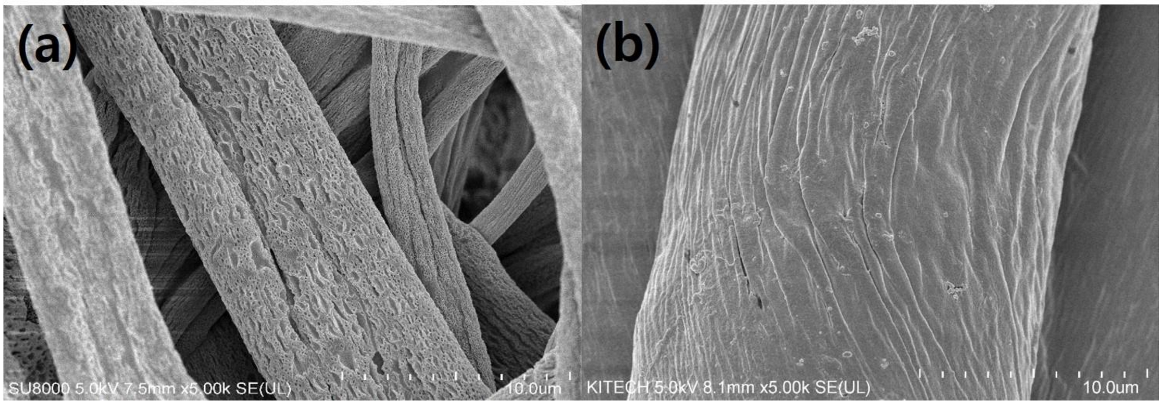

Figure 1. SEM images showing morphologies of: (a) curcumin dyed and deacetylated cellulose acetate fiber; and (b) curcumin dyed cotton fiber.

The fastness property of CECF was examined upon washing the CECF with ethanol and water. The CECF was immersed in ethanol, and subsequently immersed in water. The washing process was repeated three times. The repetitive washing with protic solvents did not significantly change the color of CECF and sensing behavior. The color preservation and SEM images of CECF and washed CECF are presented in Figure S2.

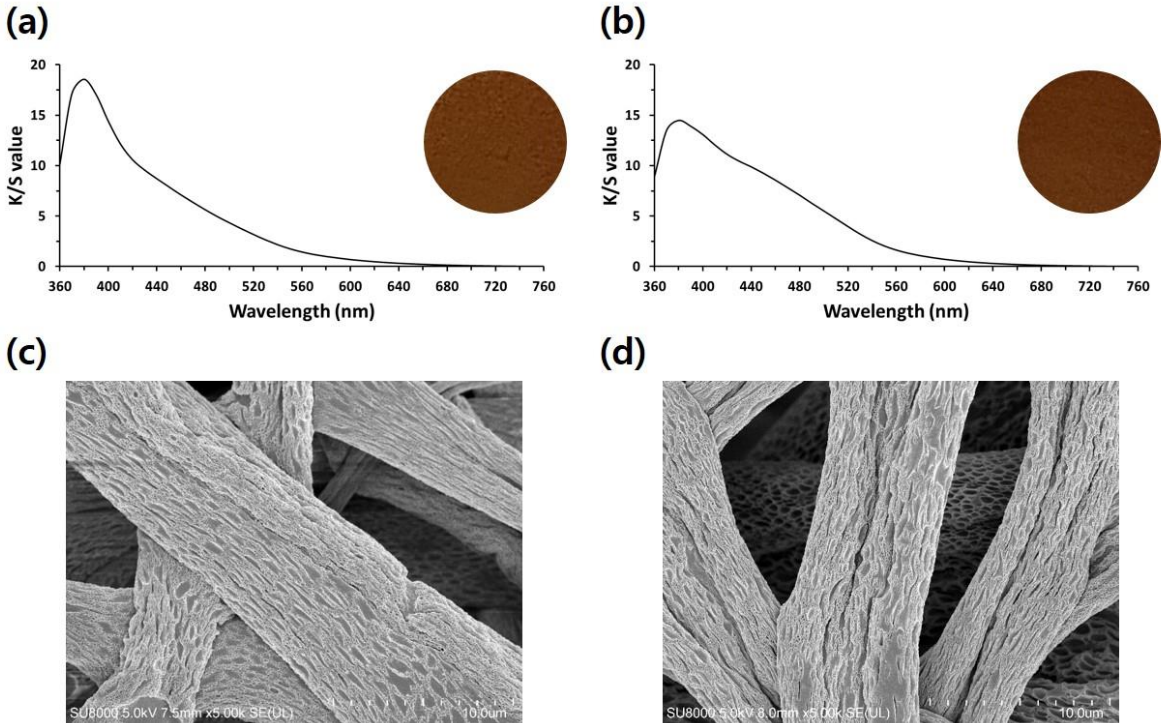

Figure S2. Plots showing K/S value as a function of wavelength of (a) the CECF and (b) the CECF after washing, respectively (inset images are the photographs confirming the color), and SEM images of (c) the CECF and (d) the CECF after washing, respectively.

The CECF exposed HCl vapor presents clear yellow color, as shown in Figure S3a. The yellow color was partially changed to yellow-brown color after washing process. The changed color completely returned back to yellow color after re-exposure to HCl vapor, strongly suggesting that the curcumin molecules on the surface were strongly adsorbed on the surface of the CECF. The SEM image presents any significant change of morphology.

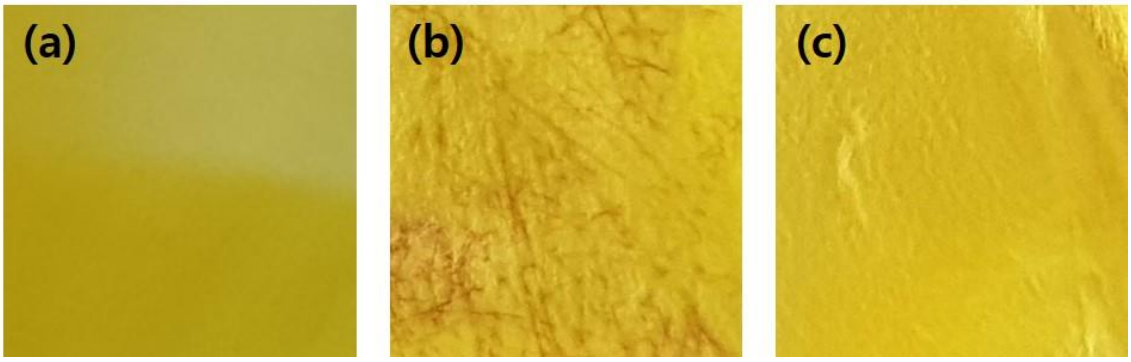

Figure 2. Photographs showing fastness tests of: (a) a CECF after HCl exposure; (b) after washing process of the CECF after HCl exposure; and (c) re-exposed washed CECF to HCl vapor.

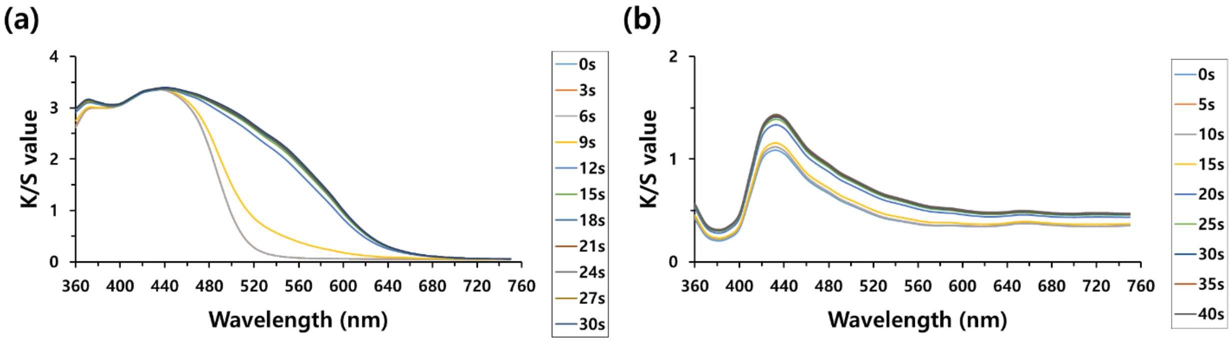

Figure 3. Plots showing the changes of K/S value upon the exposure of samples to ammonia gas: (a) CECF; and (b) dyed cotton.

© 2020 by the authors. Submitted for possible open access publication under the terms and conditions of the Creative Commons Attribution (CC BY) license (http://creativecommons.org/licenses/by/4.0/).
